# Electroencephalography Correlation of Ketamine-induced Clinical Excitatory Movements: A Systematic Review

**DOI:** 10.5811/westjem.18611

**Published:** 2024-11-21

**Authors:** Emine M. Tunc, Neil Uspal, Lindsey Morgan, Sue L. Groshong, Julie C. Brown

**Affiliations:** * University of Texas Southwestern, Department of Pediatrics, Division of Emergency Medicine, Dallas, Texas; † University of Washington, Department of Pediatrics, Division of Emergency Medicine, Seattle, Washington; ‡ University of Washington, Department of Pediatrics, Division of Neurology, Seattle, Washington; § Seattle Children’s Hospital and Research Institute, Library and Information Commons, Seattle, Washington

## Abstract

**Background:**

This is a systematic review investigating the correlation between seizures identifiable on electroencephalogram (EEG), clinical excitatory movements (CEM), and ketamine administration for procedural sedation.

**Methods:**

We searched MEDLINE, EMBASE, Cochrane CENTRAL, and Web of Science in April 2021. Search terms included variations for ketamine, myoclonus, seizures, status epilepticus, and electroencephalography. Two independent reviewers assessed papers based on eligibility criteria, which included human studies where EEG recordings were obtained during ketamine administration.

**Results:**

Eight papers were eligible for inclusion with 141 subjects (24 children). Seven studies (133 subjects) reported epilepsy history; 70% (94/133) of these subjects had a pre-existing epilepsy diagnosis. No (0/39) subjects without epilepsy and 28% (26/94) of subjects with epilepsy had electrographic seizures after ketamine administration. In four studies where pediatric and adult subjects could be separated, children with epilepsy had electrographic seizures in 60% (3/5) of cases compared to 28% (6/33) of cases of adults with epilepsy. Of the subjects with epilepsy, 14% (10/74) had CEMs vs 5% (1/21) in subjects without epilepsy. Most CEMs (9/11) were temporally correlated with electrographic seizures.

**Conclusions:**

Our findings indicate that in subjects with epilepsy, electrographic seizures were frequently seen with ketamine administration and were correlated with CEMs. No seizure activity after ketamine was seen in subjects without epilepsy. While the clinical significance of these findings needs further investigation, clinicians may want to consider patients’ seizure history when providing counseling on the risks and benefits of ketamine sedation.

## INTRODUCTION

### Background

Ketamine is one of the most used anesthetic medications for procedural sedation. In children, ketamine is the sedative of choice in up to 80% of children in the emergency department (ED),^1^ while in adults ketamine is gaining increasing favor based on its desirable safety profile.^2^ It is a dissociative anesthetic that creates a perception of detachment from environment and self.^3^ Low rates of respiratory and cardiovascular adverse events make ketamine a favorable preferred for procedural sedation compared to other popular anesthetic medications.^1^ ^,^ ^2^

One of the associated side effects of ketamine, recognized since it was first discovered in 1960s, are excitatory movements such as twitching and hypertonicity.^4^ In 2009, based on the consensus guidelines on reporting adverse events during procedural sedation, these movements were termed as clinical excitatory movements (CEM) and classified into three groups: myoclonus; muscle rigidity; and generalized motor seizures.^5^ Most CEMs are of short duration, but even when self-limited these movements may cause distress to caregivers or staff as they resemble seizures. It is unclear whether CEMs are epileptic or are unrelated to seizure activity. Given this ambiguity, it is important to understand the risk of seizures with ketamine administration such that clinicians can better weigh the risks and benefits of this medication.

The underlying etiologies of CEMs are unknown. To determine whether CEMs are seizure related, a concurrent electroencephalogram (EEG) is necessary, particularly as sedation may alter distinguishing epileptic features such as pupillary changes or eye movements (ie, nystagmus), alterations in mentation, or motor manifestations.^6^ We conducted a systematic review to answer two questions: 1) Does ketamine induce electrographic seizures; and 2) are ketamine-induced CEMs associated with electrographic seizures with special attention to pediatric subjects?

## METHODS

### Study Design

We followed the Preferred Reporting Items for Systematic Reviews and Meta-Analyses (PRISMA) guidelines for this systematic review.^7^ A search strategy was developed in conjunction with a medical librarian. We searched Ovid MEDLINE (1946 to April 27, 2021), Elsevier EMBASE (1974 to April 2021), Cochrane Central Register of Controlled Trials (CENTRAL) (*The Cochrane Library*, Issue 3 of 12, March 2021) and Web of Science Core Collection, Science Citation Index (1985 to April 2021). The MEDLINE search was performed using Medical Subject Headings and text words for ketamine, myoclonus, seizures, status epilepticus, and EEG. The MEDLINE strategy was adapted to search the other databases. Results were limited to English-language publications. Inclusion criteria were the use of human study subjects and the use of EEG testing during ketamine administration. We excluded studies using ketamine for patients with ongoing seizures. Comments, editorials, letters, notes, and conference abstracts were excluded in MEDLINE and EMBASE. The details of the search strategy can be accessed in Supplement 1.

Records were screened by title and abstract, and all potentially relevant papers were obtained for full-text review. Full-text papers were included based on the inclusion criteria. We included additional reports based on review of included paper citations. Each manuscript was abstracted by investigators ET and NU independently, and discrepancies were resolved by consensus.

### Outcome Measures

There were two primary outcomes: 1) the frequency of electrographic seizures recorded on surface and/or deep electrodes following ketamine administration (excluding studies using ketamine for patients with ongoing seizures) and 2) the prevalence of concurrence of ketamine-induced CEMs with electrographic seizures.

We identified electrographic seizures as EEG recordings with concurrent electrographic seizure activity, as defined by the EEG definition at the time of the paper publication. The EEG recordings were rarely available in the manuscripts and, when available, only included a few seconds of the recording. Thus, EEGs were considered positive for seizures based on the authors’ report. A variety of ketamine-induced CEMs were reported that included twitching, myoclonic jerks, extremity tonic movements, generalized tonic-clonic movements, and major motor convulsions. Increased muscle tone or orofacial dyskinesias (nystagmus, tongue fasciculations) were not considered CEMs.

We defined subjects as children younger than 18 years of age.

## RESULTS

### Literature Search

The initial literature search resulted in 583 individual records. After reviewing titles and abstracts, 20 potentially eligible papers qualified for full-text review. Review of the citations for the 20 reviewed papers resulted in an additional five potentially eligible papers, which were also retrieved for full text review. After the review of all 25 full-text papers, eight^8^ ^–^ ^15^ were included in the study (Figure).

**Figure.**
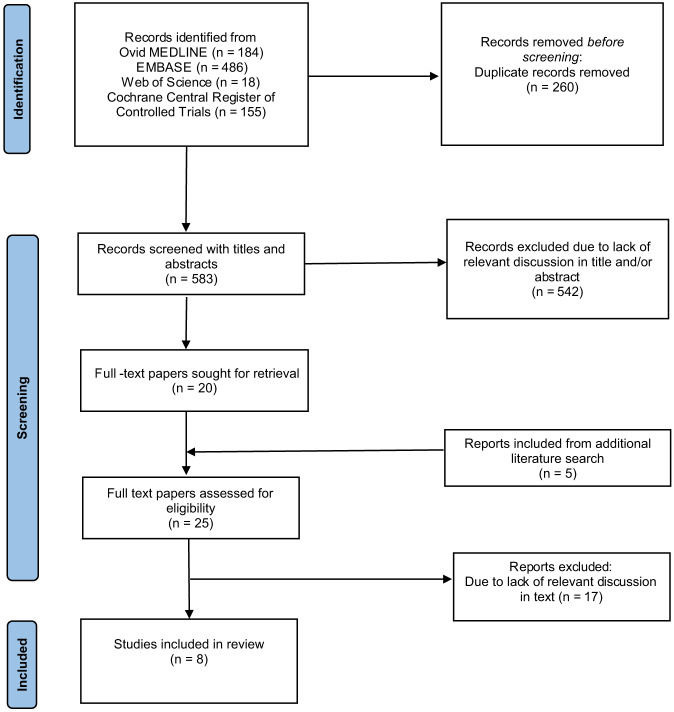
Flow of study selection reported according to PRISMA 2020 guidelines.

### Study Characteristics

These eight studies encompassed 141 subjects, including 24 children. In four^8^ ^–^ ^10^ ^,^ ^14^ of the studies (involving 12 adults and 24 children) ketamine was used for procedural sedation; in three^12^ ^,^ ^13^ ^,^ ^15^ studies (involving 56 adults and 40 subjects whose sex was not specified), ketamine was administered to volunteers for research purposes only, and in one^11^ study of 30 adults, the purpose was not explicitly stated. Six of eight studies were specifically designed to assess ketamine’s effect on electrographic seizures.^8^ ^,^ ^9^ ^,^ ^12^ ^–^ ^15^ All studies used conventional EEG with surface electrodes. Surface electrodes were placed to the skull in the international 10–20 EEG distribution. In one study with nine epileptic subjects in addition to surface electrodes, deep electrodes were surgically implanted “bilaterally to limbic region (amygdala), hippocampus (anterior, middle and posterior pes), and hippocampal gyrus (anterior, middle and posterior gyrus).” The deep electrodes recorded electrographic seizures in all subjects receiving more than 2 milligrams per kilogram (mg/kg) ketamine; however, this activity did not spread to the cortex, as surface electrodes did not record any electrographic seizures^9^ (Table 1).

**Table 1.** Characteristics of study subjects.

Author, year	Total subjects (n)	Age range (years)	Pediatric subjects (%)	Subjects with epilepsy (%)	Indication of ketamine	Route	Dose (mg/kg per dose)	Number of doses	EEG type	Electrographic seizures (n/total)	Clinical excitatory movements
Corssen, 1969^8^	11	3–13	100	0	Elective surgery	IV	2.2	2	Surface	0/11	1 /11
Bennett, 1973^9^	8	5–27	50	100	Dental procedure	Initial IM, then IV	IM - 6.5–13 IV - 1–4.5	1 to 6	Surface	3/8	2/8^a^
Ferrer-Allado, 1973^10^	9	17–37	11	100	Localization of seizure focus	IV	1/9 – 0.5 2/9 −1 4/9 – 2 2/9 – 4	1	Surface deep (simultaneously recorded)	Surface electrodes: 0/9 deep electrodes with 0.5 mg/kg: 1/1 with 1 mg/kg: 0/2 with > = 2 mg/kg: 6/6	0/3 at ≤ 1 mg/kg 2/4^a^ at 2 mg/kg 1/2^a^ at 4 mg/kg
Schwartz, 1974^11^	9	18–56	0	0	Unknown	IV	2	2	Surface	0/9	Unknown
Corssen, 1974^12^	30	19–68	0	70	Research	IV	2.2	1	Surface	With epilepsy: 1/21 increased from baseline without epilepsy: 0/9	Unknown
Celesia, 1975^13^	26	17–58	Unknown	100	Unknown	IV	4 pts-0.5 followed by 1 22 pts- 2	4 pts 2 22 pts 1	Surface	8/26 (15/26 subjects had while asleep)	1/26
Rosen, 1976^14^ b	8	Unknown	100	Unknown	Procedural sedation	IM	5–15	1	Surface	1/8	Unknown
Venkataraman, 1983^15^	40	Unknown	Unknown	75	Research	IV	2 followed by 1	2	Surface	With epilepsy: 8/30 without epilepsy: 0/10	4/30^a^ with epilepsy

a Clinical excitatory movements associated with electroencephalography seizures.

b Not included in Table 2 due to unknown epilepsy status.

*EEG*, electroencephalography; *IM*, intramuscular; *IV*, intravenous; *mg/kg*, milligrams per kilogram.

Four studies provided age data such that pediatric (16 in three studies) and adult subjects (63 in four studies) could be reported separately.^9^ ^–^ ^12^ The age range for pediatric subjects in these studies was 3–17 years.

### Electrographic Seizures

Data on electrographic seizures were reported for all subjects. All but one study^14^ provided information on subjects’ epilepsy status (Table 1). Seventy percent of the subjects (94/133) had a diagnosis of epilepsy. Only subjects with epilepsy (28%, 26/94) had electrographic seizures. This was observed for both adult and pediatric subjects (Table 2). One fourth of those seizures (7/26) were recorded on deep electrodes where simultaneous surface EEG did not show electrographic seizures. Duration of electrographic seizures was seldom reported, but in the five cases in which duration was provided it ranged from 20 seconds to 3.5 minutes.^9^ ^,^ ^15^

**Table 2.** Summary of electrographic seizures.

	Subjects with epilepsy	Subjects without epilepsy
All subjects		
Number of subjects	94	39
Electrographic seizures	26 (28%)	0
Adult subjects^a^		
Number of subjects	33	30
Electrographic seizure	6 (28%)	0
Pediatric subjects^b^		
Number of subjects	5	11
Electrographic seizures	3 (60%)	0

*EEG*, electroencephalography.

a Included papers: Bennett 1973,^9^ Ferrer 1973,^10^ Schwartz 1974,^11^ Corssen 1974.^12^

b Included papers: Corssen 1969,^8^ Bennett 1973,^9^ Ferrer 1973.^10^

### Clinical Excitatory Movements

The presence or absence of CEMs was reported for 94 subjects (66.7%). The CEMs were more common in subjects with epilepsy (14% 10/24) than subjects without epilepsy (5%, 1/21). The subjects who had both CEMs and electrographic seizures were reviewed for the type, duration, and quality of their events (Tables 3 and 4). Types of CEMs were either focal motor movements or generalized tonic-clonic motor activity, and rarely myoclonus or jerking movements. In the 11 subjects with CEMs, 9 had temporally correlating electrographic seizures, and all these patients had a history of epilepsy. Four of the aforementioned patients with electrographic seizures were identified on deep-electrode recordings only, and not on the simultaneous recording with surface electrodes. Only one patient without epilepsy was described as having CEMs, and they were not associated with epileptiform discharges on the EEG recording. For the one remaining patient with CEM, there wasn’t information on correlation with EEG seizure.

**Table 3.** Summary of clinical excitatory movements.

	Subjects with epilepsy	Subjects without epilepsy
All subjects		
Number of subjects	73	21
Clinical excitatory movements		
Positive	10^a^ (14%)	1 (0.5%)
Negative	63 (86%)	20 (99.5%)
Adult subjects^b^		
Number of subjects	12	12
Clinical excitatory movements		
Positive	3^d^ (25%)	0
Negative	9 (75%)	12 (100%)
Pediatric subjects^c^		
Number of subjects	5	11
Clinical excitatory movements		
Positive	2^d^ (40%)	1 (9%)
Negative	3 (60%)	10 (81%)

a Nine subjects also had electrographic seizures.

b Included papers: Bennett 1973,^9^ Ferrer 1973.^10^

c Included papers: Corssen 1969,^8^ Bennett 1973,^9^ Ferrer 1973.^10^

d Clinical excitatory movements associated with electroencephalography seizures.

**Table 4.** Subjects with clinical excitatory movements.

	Age	Electrographic seizures immediately after ketamine administration	Type of CEM	Baseline EEG	Baseline seizure semiology
Corssen, 1969^8^				
No ID	<13	None	Twitching of the arms and legs	N/A	None
Bennett, 1973^9^				
Case 2	27	Polyspike and wave discharges, maximal over the left anterior temporal region (increase in baseline EEG discharges)*	Brief clonic movements of the right hand and face followed by 1 minute right tonic [adversive] seizure	Slow posterior rhythms, decreased amplitude over left temporal area, left frontotemporal spikes and sharp and slow waves	Focal and generalized motor seizures
Case 7	17	Right temporal focal discharges (different than baseline discharges)*	Left tonic [adversive] seizure	Left temporal spike and slow waves with secondary synchrony, slow posterior rhythms	Generalized motor seizures
Ferrer-Allado, 1973^10^				
Subject 1	17	Seizure activity in deep electrodes*	Tonic-clonic motor activity	Unknown	Unknown type
Subject 7	20	Seizure activity in deep electrodes*	Tonic-clonic motor activity	Unknown	Unknown type
Subject 8	33	Seizure activity in deep electrodes*	Jerking motor movements, clonic motor activity	Unknown	Unknown type
Celesia, 1975^13^				
No ID	Unknown	Unknown if the same subject had electrographic seizures	Sporadic myoclonic jerks	Unknown	Psychomotor seizures
Venkataraman, 1983^15^				
No ID	Unknown	Seizure discharges*	Tonic-clonic motor activity lasting 3 min	Unknown	Unknown type
No ID	Unknown	Seizure discharges*	Tonic-clonic motor activity lasting 3 min	Unknown	Unknown type
No ID	Unknown	Seizure discharges*	Tonic-clonic motor activity lasting 3 min	Unknown	Unknown type
No ID	Unknown	Increased seizure discharges from baseline*	Clinical seizure	Generalized spike and wave discharges	Unknown type

[] old terminology

* CEM correlated with electrographic seizures.

*CEM*, clinical excitatory movement; *EEG*, electroencephalography; *ID*, identity; *N/A*, not applicable.

There were some behavioral changes reported in two studies that were not included as CEMs in our systematic review given they were not associated with rhythmic movements. Venkataraman et al reported increased “muscle tone” in 24 and “orofacial dyskinesias” in 22 of 30 patients. Celesia et al reported “unusual postures” in 12 and “motor hyperactivity” in 5 of 26 patients. The presence of electrographic seizures in correlation with these movements were not specified in these studies.

## DISCUSSION

We found that in subjects with epilepsy there is some evidence of ketamine provoking electrographic seizures. In subjects without epilepsy, no electrographic seizures were seen with ketamine administration. Most of the subjects who had CEMs also had temporally correlated electrographic seizures. Given the infrequency of myoclonic jerks observed in this study, it remains unclear whether this activity may be epileptiform. This relationship between ketamine and electrographic seizures may be dose dependent. In Ferrer-Allado et al the patients with electrographic seizures were given higher doses (2 and 4 mg/kg) of ketamine rather than the more typical induction doses of 1–2 mg/kg used at the start of procedural sedation.^10^ ^,^ ^16^ ^,^ ^17^

There is a growing body of literature on the use of ketamine for refractory status epilepticus (RSE) in adults and children. Ketamine has a reported efficacy in stopping seizures of up to 73% for adult and 74% for pediatric patients.^18^ Although the mechanism of action is not exactly known, it is hypothesized that ketamine as a non-competitive antagonist of N-methyl-D-aspartate (NMDA) receptors can deactivate NMDA receptors that are activated by glutamate overflow during RSE. Considering ketamine’s documented antiseizure effects, it would be unexpected for ketamine to additionally be associated with epileptogenic activity. Our primary outcome was to evaluate EEG changes following ketamine administration during the neutral state of the brain.

One could speculate that a neutral/non-excitatory state of the brain may respond to ketamine differently than the brain in the excitatory state that is seen during RSE. There is evidence in animal studies that as seizures prolong, numbers of NMDA receptors increase, and gamma-aminobutyric acid receptors decrease on the postsynaptic surface. This changing cellular structure might play a role in the effectiveness of ketamine, an NMDA receptor antagonist, in halting RSE.^19^ Another possible explanation is that observed electrographic seizures in subjects with epilepsy during ketamine may be confounded by the frequency of electrographic seizures at baseline in these subjects. However, described electrographic seizures with ketamine were not always identical with baseline epileptiform discharges, which may suggest that these were separate electrographic seizures provoked by ketamine.^9^ ^,^ ^10^ ^,^ ^12^

The association of seizures and decreased cognitive function has long been recognized.^20^ These effects have mainly been shown with prolonged seizures or epilepsy disorders.^20^ ^,^ ^21^ The effects of a single seizure on cognition in children seem to be insignificant, whereas effects in adults are unclear.^22^ ^,^ ^23^ Furthermore, for electrographic-only seizures, data suggests that high seizure burden is required to cause clinical impact.^22^ Thus, even though there is some evidence that ketamine may be provoking brief electrographic seizures, the effect of these seizures on cognition is likely not clinically significant.

Seven of 26 subjects who had electrographic seizures were recorded via deep electrodes that were not captured by surface electrodes. Similarly, studies using deep electrodes in animals with epilepsy have also shown electrographic seizures during ketamine administration,^24^ ^,^ ^25^ whereas animals without history of epilepsy did not show any electrographic seizures.^26^ The involvement of subcortical structures in modulation and propagation of seizures has been described; however, the incidence and clinical significance of the deep electrographic seizures is unknown compared to surface electrographic seizures.^27^

## LIMITATIONS

There are several limitations of this systematic review. Because this study was limited to English-language studies, we could have missed studies in other languages. The number of subjects in each study was small, and there were differences in design and patient populations between studies. In only three of the studies was ketamine used for procedural sedation; in the other studies ketamine was administered to volunteers, which may limit generalization to ketamine use in procedural sedation. The eligible studies were performed between 40–50 years ago when EEG capabilities were less advanced than today.^28^ ^,^ ^29^ Finally, most of the EEG recordings described in the studies were not available for review. The CEMs were described in generalized terms, and exact semiology often was not described.

## CONCLUSION

This is the first systematic review to document the relationship between ketamine and clinical excitatory movements. The limited available data is insufficient to make strong conclusions on the risk and clinical significance of seizures with ketamine, and the correlation of CEMs with electrographic seizure. All observed seizures were brief, with none meeting the definition of status epilepticus.^30^ While CEMs correspond to electrographic seizure in patients with a history of seizures, it is unclear whether this is clinically important. Based on our findings, we recommend that clinicians who administer sedation ask about a patient’s history of epilepsy during their pre-sedation assessment to inform their assessment of the risks and benefits of ketamine sedation and discuss the potential risk of increased electrographic seizures. Further studies, especially with video-EEG monitoring during ketamine sedation in epilepsy patients, are needed.

## Supplementary Information




