# Supplementary material for: Electroencephalography Correlation of Ketamine-induced Clinical Excitatory Movements: A Systematic Review
Source: West J Emerg Med. 2024 Nov 21;26(1):147–54. doi: 10.5811/westjem.18611 (PMC11908518; doi:10.5811/westjem.18611)
Supplement: Supplementary file 1 [file wjem-26-147-s001.docx]

| **Supplementary Table 1 – Summary of clinical excitatory movements** | | |
| --- | --- | --- |
|  | Subjects with Epilepsy | Subjects without epilepsy |
| **All subjects** |  |  |
| Number of subjects | 73 | 21 |
| Clinical excitatory movements |  |  |
| Positive | 10 ^a^(14%) | 1 (0.5%) |
| Negative | 63 (86%) | 20 (99.5 %) |
| **Adult subjects^b^** |  |  |
| Number of subjects | 12 | 12 |
| Clinical excitatory movements |  |  |
| Positive | 3^d^ (25 %) | 0 |
| Negative | 9 (75 %) | 12 (100 %) |
| **Pediatric subjects^c^** |  |  |
| Number of subjects | 5 | 11 |
| Clinical excitatory movements |  |  |
| Positive | 2^d^ (40 %) | 1 (9 %) |
| Negative | 3 (60 %) | 10 (81 %) |
| ^a^Nine subjects also had electrographic seizures.  ^b^Included articles: Bennett 1973, Ferrer 1973  ^c^Included articles: Corssen 1969, Bennett 1973, Ferrer 1973  ^d^Clinical excitatory movements associated with electroencephalography seizures | | |

| **Supplementary Table 2: Subjects with clinical excitatory movements** | | | | | | | | |  |  |
| --- | --- | --- | --- | --- | --- | --- | --- | --- | --- | --- |
|  | **Age** | | **Electrographic Seizures Immediately After Ketamine Administration** | | **Type of CEM** | **Baseline EEG** | **Baseline Seizure Semiology** |  |  |  |
| **Corssen, 1969**^6^ | | | |  | | | | |  |  |
| No ID | <13 | | None | | Twitching of the arms and legs | N/A | None |  |  |  |
|  |  | |  | |  |  |  |  |  |  |
| **Bennett, 1973**^7^ | | | |  | | | | |  |  |
|  | | | |  | | | | |  |  |
| Case 2 | | 2 7 | Polyspike and wave discharges, maximal over the left anterior temporal region(increase in baseline EEG discharges)* | | Brief clonic movements of the right hand and face followed by 1 minute right tonic [adversive] seizure | Slow posterior rhythms, decreased amplitude over left temporal area, left frontotemporal spikes and sharp and slow waves | Focal and generalized motor seizures |  |  |  |
| Case 7 | | 17 | Right temporal focal discharges(different than baseline discharges)* | | Left tonic [adversive] seizure | Left temporal spike and slow waves with secondary synchrony, slow posterior rhythms | Generalized motor seizures |  |  |  |
|  | |  |  | |  |  |  |  |  |  |
| **Ferrer-Allado,**  **1973**^8^ | | | | |  |  |  |  |  |  |
| Subject 1 | | 17 | Seizure activity in deep electrodes* | | Tonic-clonic motor activity | Unknown | Unknown type |  |  |  |
| Subject 7 | | 20 | Seizure activity in deep electrodes* | | Tonic-clonic motor activity | Unknown | Unknown type |  |  |  |
| Subject 8 | | 33 | Seizure activity in deep electrodes* | | Jerking motor movements, clonic motor activity | Unknown | Unknown type |  |  |  |
|  | |  |  | |  |  |  |  |  |  |
| **Celesia, 1975**^11^ | | | |  | | | | |  |  |
| No ID | Unkown | | Unknown if the same subject had electrographic seizures | | Sporadic myoclonic jerks | Unknown | Psychomotor seizures |  |  |  |
|  |  | |  | |  |  |  |  |  |  |
| **Venkataraman, 1983**^13^ | | | |  | | | | |  |  |
| No ID | Unknown | | Seizure discharges* | | Tonic-clonic motor activity lasting 3 min | Unknown | Unknown type |  |  |  |
| No ID | Unknown | | Seizure discharges* | | Tonic-clonic motor activity lasting 3 min | Unknown | Unknown type |  |  |  |
| No ID | Unknown | | Seizure discharges* | | Tonic-clonic motor activity lasting 3 min | Unknown | Unknown type |  |  |  |
| No ID | Unknown | | Increased seizure discharges from baseline* | | Clinical seizure | Generalized spike and wave discharges | Unknown type |  |  |  |
| CEM: Clinical excitatory movement, EEG: Electroencephalography, ID: identity, N/A: Not applicable, []old terminology  *CEM correlated with electrographic seizures | | | | | | | | |  |  |

|  | **Literature Search Summary** |
| --- | --- |

.

Contents

[Databases Searched 2](#_Toc70515190)

[Question 2](#_Toc70515191)

[Search Summary 2](#_Toc70515192)

[Results 2](#_Toc70515193)

[Search Strategies 2](#_Toc70515194)

# Databases Searched

| **Database** | **Date Searched** | **Results** |
| --- | --- | --- |
| Ovid MEDLINE(R) and Epub Ahead of Print, In-Process & Other Non-Indexed Citations, Daily and Versions(R) (1946 to April 27, 2021) | 4/28/21 | 185 |
| EMBASE.com (1974 to Present, includes Medline 1966 to Present) | 4/28/21 | 486 |
| Cochrane Central Register of Controlled Trials (CENTRAL, *The Cochrane Library*, Issue 3 of 12, March 2021) | 4/28/21 | 18 |
| Web of Science Core Collection (Science Citation Index Expanded, 1985 to Present) | 4/28/21 | 155 |
| **TOTAL** | | **844** |

# Question

P: primary population 0-18 years old, secondary population >18 years old, animals (rat, primate)

I: Administration of ketamine (IV and IM)

C: versus no sedation (OR other sedations)

O: primary outcome clinical excitatory movements (this will include myoclonus, jerks, tonic clonic seizures, seizures) or secondary outcome EEG detectable seizures

# Search Summary

We searched Ovid MEDLINE (1946 to April 27, 2021 with Epub Ahead of Print, In-Process and Other Non-Indexed Citations), EMBASE.com (1974 to April 2021), Cochrane Central Register of Controlled Trials (CENTRAL) (*The Cochrane Library*, Issue 3 of 12, March 2021) and Web of Science (1985 to April 2021).

The MEDLINE search was performed using MeSH and text words for ketamine, myoclonus, seizures, status epilepticus and electroencephalography. The MEDLINE strategy was adapted to search EMBASE, CENTRAL and Web of Science. Results were further limited to English language. Specified publication types were excluded in MEDLINE and EMBASE (e.g., comments, editorials, letters, notes, conference abstracts).

# Results

Results were returned as an Excel file.

# Search Strategies

Ovid Medline

1 Ketamine/

2 Analgesics/ and 19660101:19711231.(da). [previous indexing 1966-1971]

3 Anesthetics/ and 19720101:19721231.(da). [previous indexing 1972]

4 Cyclohexanes/ and 19680101:19721231.(da). [previous indexing 1968-1972]

5 ("1867-66-9" or "6740-88-1" or "81771-21-3" or anesject or calipsol or calypsol or imalgene or kalipsol or katamine or keta hameln or ketaject or ketalar or ketalin or ketamax or ketamine or ketaminol vet or ketanest or ketased or ketaset or ketaved or ketavet or ketmin or ketoject or ketolar or narkamon or narketan or soon soon or tekam or velonarcon or vetalar).tw,kf. [title, abstract, author kw]

6 ketamine.nm. or ("690g0d6v8h" or "o18yuo0i83").tw,kf,rn. [name of substance, title, abstract, author kw, registry number]

7 or/1-6 [Ketamine]

8 Myoclonus/

9 Epilepsies, Myoclonic/ and 19920101:19991231.(da). [Myoclonic Jerking 1992-1999]

10 Spasms, Infantile/ and 19770101:19991231.(da). [Massive Myoclonic Jerking 1977-1999]

11 Seizures/ or Status Epilepticus/

12 Epilepsy/ and 19660101:19781231.(da). [previous indexing 1966-1978]

13 (contract* or convuls* or jerk* or myoclon* or paramyoclon* or polymyoclon* or seiz* or spasm* or twitch*).tw,kf. [title, abstract, author kw]

14 (absence status or continu* partial* epilep* or epilep* partial* continu* or epileptic status or partial* contin* epilep* or petit mal status or simple partial status or status epileptic*).tw,kf. [title, abstract, author kw]

15 ((kojevnikoff* or kojevnikov* or kojewnikofzure* or kojewnikoff* or kojewnikov* or kojewnikow* or koshernikoff* or kozhevnikov*) adj2 (epilep* or syndrome*)).tw,kf. [title, abstract, author kw]

16 ((continu* or incontrol* or prolong* or uncontrol*) adj3 seiz*).tw,kf. [title, abstract, author kw]

17 or/8-16 [Myoclonus/Seizures]

18 exp Brain Waves/ or Electroencephalography/

19 (brainwave* or brain wave* or eeg or electroencephalogra* or elect* encephalogra*).tw,kf. [title, abstract, author kw]

20 ((alpha or beta or delta or gamma or theta) adj2 (activit* or rhythm* or wave*)).tw,kf. [title, abstract, author kw]

21 (brain* adj3 activ*).tw,kf. [title, abstract, author kw]

22 or/18-21 [EEG]

23 (comment or editorial or letter or news).pt.

24 (7 and 17 and 22 and english.lg.) not 23

Embase

#16 #15 AND [english]/lim NOT ('conference abstract'/it OR 'conference review'/it OR editorial/it OR letter/it OR note/it)

#15 #3 AND #10 AND #14

#14 #11 OR #12 OR #13

#13 ((alpha OR beta OR delta OR gamma OR theta) NEAR/2 (activit* OR rhythm* OR wave*)):ti,ab,kw

#12 ((brain NEAR/3 activ*):ti,ab,kw) OR brainwave*:ti,ab,kw OR ((brain NEXT/1 wave*):ti,ab,kw) OR 'eeg':ti,ab,kw OR electroencephalogra*:ti,ab,kw OR ((elect* NEXT/1 encephalogra*):ti,ab,kw)

#11 'electroencephalography'/de OR 'electroencephalography monitoring'/de OR 'electroencephalogram'/exp OR 'electroencephalograph'/exp OR 'eeg abnormality'/de

#10 #4 OR #5 OR #6 OR #7 OR #8 OR #9

#9 ((continu* OR incontrol* OR prolong* OR uncontrol*) NEAR/3 seiz*):ti,ab,kw

#8 ((kojevnikoff* OR kojevnikov* OR kojewnikofzure* OR kojewnikoff* OR kojewnikov* OR kojewnikow* OR koshernikoff* OR kozhevnikov*) NEAR/2 (epilep* OR syndrome*)):ti,ab,kw

#7 'absence status':ti,ab,kw OR ((continu* NEXT/1 partial* NEXT/1 epilep*):ti,ab,kw) OR ((epilep* NEXT/1 partial* NEXT/1 continu*):ti,ab,kw) OR 'epileptic status':ti,ab,kw OR ((partial* NEXT/1 contin* NEXT/1 epilep*):ti,ab,kw) OR 'petit mal status':ti,ab,kw OR 'simple partial status':ti,ab,kw OR ((status NEXT/1 epileptic*):ti,ab,kw)

#6 contract*:ti,ab,kw OR convuls*:ti,ab,kw OR jerk*:ti,ab,kw OR myoclon*:ti,ab,kw OR paramyoclon*:ti,ab,kw OR polymyoclon*:ti,ab,kw OR seiz*:ti,ab,kw OR spasm*:ti,ab,kw OR twitch*:ti,ab,kw

#5 'seizure'/de OR 'epileptic state'/de

#4 'myoclonus'/de OR 'myoclonus seizure'/de

#3 #1 OR #2

#2 anesject:ti,ab,kw OR calipsol:ti,ab,kw OR calypsol:ti,ab,kw OR imalgene:ti,ab,kw OR kalipsol:ti,ab,kw OR katamine:ti,ab,kw OR 'keta hameln':ti,ab,kw OR ketaject:ti,ab,kw OR ketalar:ti,ab,kw OR ketalin:ti,ab,kw OR ketamax:ti,ab,kw OR ketamine:ti,ab,kw OR 'ketaminol vet':ti,ab,kw OR ketanest:ti,ab,kw OR ketased:ti,ab,kw OR ketaset:ti,ab,kw OR ketaved:ti,ab,kw OR ketavet:ti,ab,kw OR ketmin:ti,ab,kw OR ketoject:ti,ab,kw OR ketolar:ti,ab,kw OR narkamon:ti,ab,kw OR narketan:ti,ab,kw OR 'soon soon':ti,ab,kw OR tekam:ti,ab,kw OR velonarcon:ti,ab,kw OR vetalar:ti,ab,kw

#1 'ketamine'/de OR '1867-66-9':rn OR '6740-88-1':rn OR '81771-21-3':rn

CENTRAL

#1 MeSH descriptor: [Ketamine] this term only

#2 (anesject OR calipsol OR calypsol OR imalgene OR kalipsol OR katamine OR "keta hameln" OR ketaject OR ketalar OR ketalin OR ketamax OR ketamine OR "ketaminol vet" OR ketanest OR ketased OR ketaset OR ketaved OR ketavet OR ketmin OR ketoject OR ketolar OR narkamon OR narketan OR "soon soon" OR tekam OR velonarcon OR vetalar):ti,ab,kw

#3 #1 OR #2

#4 MeSH descriptor: [Myoclonus] this term only

#5 MeSH descriptor: [Seizures] this term only

#6 MeSH descriptor: [Status Epilepticus] this term only

#7 (contract* OR convuls* OR jerk* OR myoclon* OR paramyoclon* OR polymyoclon* OR seiz* OR spasm* OR twitch*):ti,ab,kw

#8 ("absence status" OR (continu* NEXT partial* NEXT epilep*) OR (epilep* NEXT partial* NEXT continu*) OR "epileptic status" OR (partial* NEXT contin* NEXT epilep*) OR "petit mal status" OR "simple partial status" OR (status NEXT epileptic*)):ti,ab,kw

#9 ((kojevnikoff* OR kojevnikov* OR kojewnikofzure* OR kojewnikoff* OR kojewnikov* OR kojewnikow* OR koshernikoff* OR kozhevnikov*) NEAR/2 (epilep* OR syndrome*)):ti,ab,kw

#10 ((continu* OR incontrol* OR prolong* OR uncontrol*) NEAR/3 seiz*):ti,ab,kw

#11 #4 OR #5 OR #6 OR #7 OR #8 OR #9 OR #10

#12 MeSH descriptor: [Brain Waves] explode all trees

#13 MeSH descriptor: [Electroencephalography] this term only

#14 (brainwave* OR (brain NEXT wave*) OR eeg OR electroencephalogra* OR (elect* NEXT encephalogra*)):ti,ab,kw

#15 ((alpha OR beta OR delta OR gamma OR theta) NEAR/2 (activit* OR rhythm* OR wave*)):ti,ab,kw

#16 (brain* NEAR/3 activ*):ti,ab,kw

#17 #12 OR #13 OR #14 OR #15 OR #16

#18 #3 AND #11 AND #17 in Trials

WOS

#1 TS=(anesject OR calipsol OR calypsol OR imalgene OR kalipsol OR katamine OR "keta hameln" OR ketaject OR ketalar OR ketalin OR ketamax OR ketamine OR "ketaminol vet" OR ketanest OR ketased OR ketaset OR ketaved OR ketavet OR ketmin OR ketoject OR ketolar OR narkamon OR narketan OR "soon soon" OR tekam OR velonarcon OR vetalar)

#2 TS=(contract* OR convuls* OR jerk* OR myoclon* OR paramyoclon* OR polymyoclon* OR seiz* OR spasm* OR twitch*)

#3 TS=("absence status" OR (continu* NEAR/0 partial* NEAR/0 epilep*) OR (epilep* NEAR/0 partial* NEAR/0 continu*) OR "epileptic status" OR (partial* NEAR/0 contin* NEAR/0 epilep*) OR "petit mal status" OR "simple partial status" OR (status NEAR/0 epileptic*))

#4 TS=((kojevnikoff* OR kojevnikov* OR kojewnikofzure* OR kojewnikoff* OR kojewnikov* OR kojewnikow* OR koshernikoff* OR kozhevnikov*) NEAR/2 (epilep* OR syndrome*))

#5 TS=((continu* OR incontrol* OR prolong* OR uncontrol*) NEAR/3 seiz*)

#6 #5 OR #4 OR #3 OR #2

#7 TS=(brainwave* OR (brain NEAR/0 wave*) OR eeg OR electroencephalogra* OR (elect* NEAR/0 encephalogra*))

#8 TS=((alpha OR beta OR delta OR gamma OR theta) NEAR/2 (activit* OR rhythm* OR wave*))

#9 TS=(brain* NEAR/3 activ*)

#10 #9 OR #8 OR #7

#11 (#10 AND #6 AND #1) AND LANGUAGE: (English)

Indexes=SCI-EXPANDED Timespan=1985-2021
